# Animal Reservoir, Natural and Socioeconomic Variations and the Transmission of Hemorrhagic Fever with Renal Syndrome in Chenzhou, China, 2006–2010

**DOI:** 10.1371/journal.pntd.0002615

**Published:** 2014-01-09

**Authors:** Hong Xiao, Huai-Yu Tian, Li-Dong Gao, Hai-Ning Liu, Liang-Song Duan, Nicole Basta, Bernard Cazelles, Xiu-Jun Li, Xiao-Ling Lin, Hong-Wei Wu, Bi-Yun Chen, Hui-Suo Yang, Bing Xu, Bryan Grenfell

**Affiliations:** 1 College of Resources and Environment Science, Hunan Normal University, Changsha, China; 2 College of Global Change and Earth System Science, Beijing Normal University, Beijing, China; 3 School of Environment, Tsinghua University, Beijing, China; 4 Hunan Provincial Center for Disease Control and Prevention, Changsha, China; 5 Chenzhou Municipal Center for Disease Control and Prevention, Chenzhou, China; 6 Department of Ecology and Evolutionary Biology, Princeton University, Princeton, New Jersey, United States of America; 7 Fogarty International Center, National Institutes of Health, Bethesda, Maryland, United States of America; 8 Ecologie and Evolution, UMR 7625, UPMC-ENS, Paris, France; 9 UMMISCO UMI 209 IRD - UPMC, Bondy, France; 10 School of Public Health, Shandong University, Jinan, China; 11 Center for Disease Control and Prevention of Beijing Military Region, Beijing, China; Technical University of Mombasa, Kenya

## Abstract

**Background:**

China has the highest incidence of hemorrhagic fever with renal syndrome (HFRS) worldwide. Reported cases account for 90% of the total number of global cases. By 2010, approximately 1.4 million HFRS cases had been reported in China. This study aimed to explore the effect of the rodent reservoir, and natural and socioeconomic variables, on the transmission pattern of HFRS.

**Methodology/Principal Findings:**

Data on monthly HFRS cases were collected from 2006 to 2010. Dynamic rodent monitoring data, normalized difference vegetation index (NDVI) data, climate data, and socioeconomic data were also obtained. Principal component analysis was performed, and the time-lag relationships between the extracted principal components and HFRS cases were analyzed. Polynomial distributed lag (PDL) models were used to fit and forecast HFRS transmission. Four principal components were extracted. Component 1 (F1) represented rodent density, the NDVI, and monthly average temperature. Component 2 (F2) represented monthly average rainfall and monthly average relative humidity. Component 3 (F3) represented rodent density and monthly average relative humidity. The last component (F4) represented gross domestic product and the urbanization rate. F2, F3, and F4 were significantly correlated, with the monthly HFRS incidence with lags of 4 months (r = −0.289, *P*<0.05), 5 months (r = −0.523, *P*<0.001), and 0 months (r = −0.376, *P*<0.01), respectively. F1 was correlated with the monthly HFRS incidence, with a lag of 4 months (r = 0.179, *P* = 0.192). Multivariate PDL modeling revealed that the four principal components were significantly associated with the transmission of HFRS.

**Conclusions:**

The monthly trend in HFRS cases was significantly associated with the local rodent reservoir, climatic factors, the NDVI, and socioeconomic conditions present during the previous months. The findings of this study may facilitate the development of early warning systems for the control and prevention of HFRS and similar diseases.

## Author Summary

Hemorrhagic fever with renal syndrome (HFRS), a rodent-borne disease caused by hantaviruses, is characterized by fever, haemorrhage, headache, back pain, abdominal pain, and acute kidney injury. China has the highest incidence of HFRS worldwide. Reported cases account for 90% of the total global cases. Approximately 1.4 million HFRS cases were reported in China between 1950 and 2010. During the same time period, >46 000 people died from HFRS, and the fatality rate was 3.29%. A great deal of interest and excitement has developed recently for understanding the role of the environment in the transmission of HFRS. Our article provides evidence that rodent density and behavior depend on natural factors. Changes in animal reservoirs may lead to the emergence of new epidemics and threats to human health. However, economic development may promote a more residential environment, which could inhibit disease transmission from animals to humans by limiting their contact. We combined data about the rodent reservoir, the natural environment, and socioeconomic factors in the model. The results will be helpful for making and prioritizing preventive measures.

## Introduction

Hemorrhagic fever with renal syndrome (HFRS) is a natural focal disease characterized by fever, hemorrhagic manifestations, and acute renal dysfunction. HFRS is mainly transmitted by rodents [1]. In China, HFRS is primarily caused by one of two types of hantaviruses, Hantaan virus (HTNV) and Seoul virus (SEOV) [2].

China has the highest incidence of HFRS worldwide. Reported cases account for 90% of the total number of global cases. Approximately 1.4 million HFRS cases were reported in China between 1950 and 2010 [3]. HFRS incidence has decreased in China in recent years. However, HFRS still causes significant morbidity and mortality and is a serious public health threat [4]. Hunan Province is one of the most highly endemic areas in China; 2670 cases of HFRS were diagnosed from 2006 to 2010. Chenzhou, a subtropical city in Hunan Province, is noted for epidemics of HFRS. During a 5-year period (2006 to 2010), 321 cases were reported in Chenzhou.

HFRS is widely transmitted from rodents to humans through contact with saliva, urine or excreta from infected rodents [5]. It is closely associated with rodent density and the virus-carrying rates of the animal hosts [6]–[9]. These factors are characterized by observable seasonal and regional variation. Variations in HFRS incidence is associated with the growth and decline of rodent population density. Human hantavirus epidemics can be accurately predicted from an analysis of the population dynamics of the rodent hosts [9]–[11]. HFRS incidence is also affected by natural environmental factors such as land use, elevation, vegetation type, crop production and area, and the El Niño-Southern Oscillation (ENSO) [12]–[15]. In particular, HFRS incidence closely correlates with meteorological factors that include temperature, rainfall, and humidity [1].

Past evidence has demonstrated that outbreaks of diseases such as schistosomiasis, malaria, tuberculosis and plague are affected by environmental factors (e.g., geography, climate, and zoology), and are affected and restricted by socioeconomic factors (e.g., social institution, economic status, and population mobility) [16]. For example, social factors, such as population mobility, have an important influence on the transmission of infectious disease. Economic development and land transformation by human activities also affect infectious disease prevalence [17]. In the midstream and downstream of China's Yangtze River, schistosomiasis incidence decreased with the development of local economic conditions. This change could be attributed to increased migration from villages to cities, resulting in a reduction in exposure to cercariae in village rice paddies [18]. The distribution and population of rodent hosts and vectors are directly affected by urbanization, deforestation, irrigation works and road construction. These changes lead to high densities of rodent populations in some areas and outbreaks of some diseases (e.g., plague and Lyme disease) [19]. Few studies have considered the relationship between HFRS and socioeconomic factors and the effect of natural and socioeconomic factors on HFRS incidence.

The aim of this study was to analyze the quantitative relationship between HFRS transmission and environmental variables, to forecast the trend in prevalence of HFRS transmission, and to reveal the transmission pattern from data on HFRS cases, rodent host populations, and environmental variables (natural variables and social variables) in Chenzhou from 2006 to 2010. Natural variables included rodent density, the normalized difference vegetation index (NDVI) for cultivated land, monthly average temperature, monthly average rainfall and monthly average relative humidity. Social variables included gross domestic product (GDP) and the urbanization rate. The results may lead to the discovery of epidemic factors that are important for control of HFRS.

## Materials and Methods

### Background and data collection

**Figure 1:**
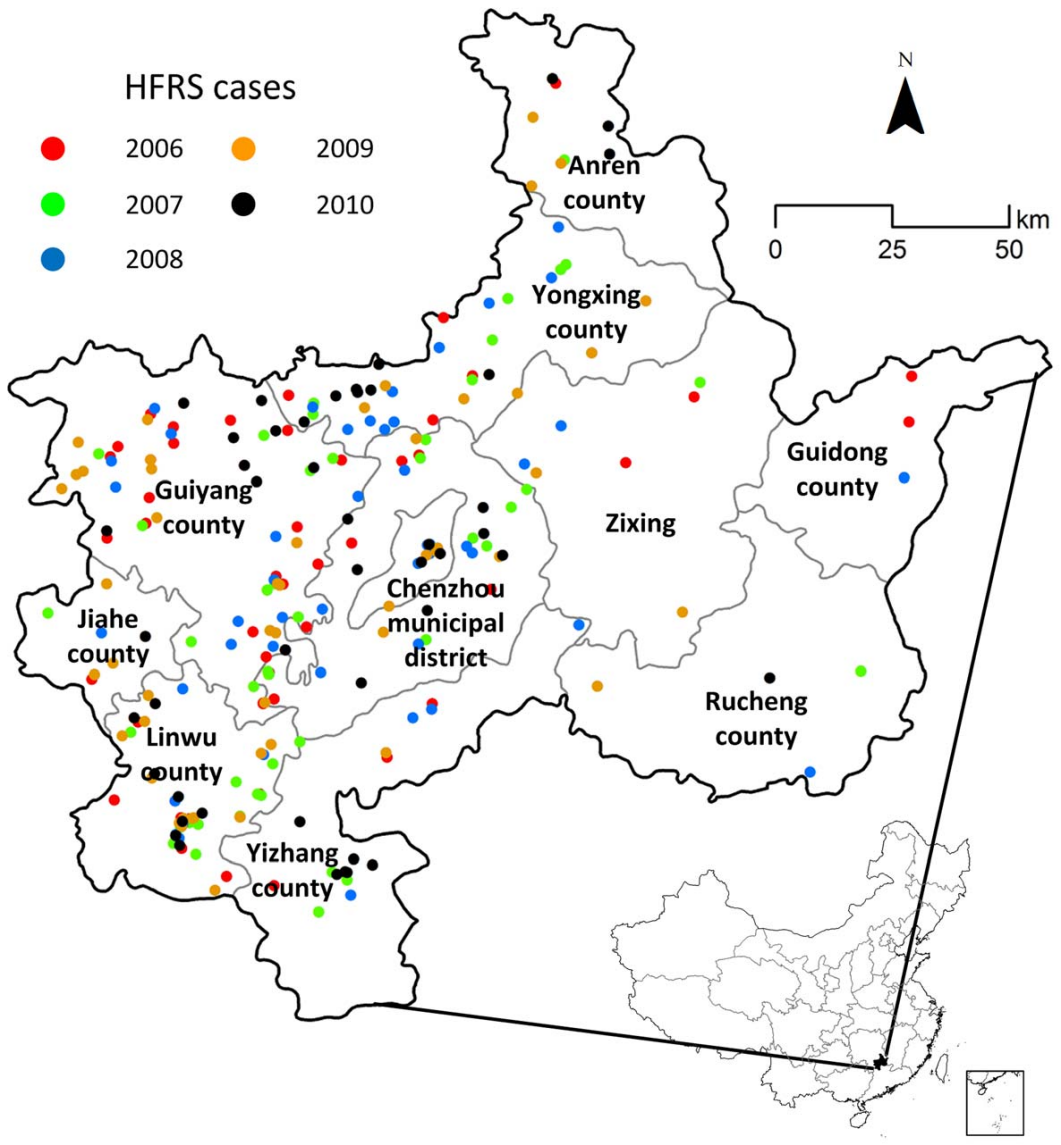
Distribution of hemorrhagic fever with renal syndrome (HFRS) cases in Chenzhou, 2006–2010.

The study area covers Chenzhou, located in a subtropical region of Hunan Province in Central China. Chenzhou is located between latitude 24°53′ and 26°50′ north, and longitude 112°13′ and 114°14′ east. It is 217 km wide and 202 km long, with a total land area of 19,400 km^2^. The region consists of two municipal districts (Beihu and Suxian) and nine counties (Guiyang, Yizhang, Yongxing, Jiahe, Linwu, Rucheng, Guidong, Anren and Zixing), and a total population of about 4.6 million people (Figure 1).

From 2006 to 2010, data from cases of HFRS in Chenzhou were obtained from the Hunan Center for Disease Control and Prevention (CDC). All of the cases were initially diagnosed based on clinical symptoms using diagnostic criteria from the Ministry of Health of the People's Republic of China. Blood samples were collected for serologic identification from all suspect cases. Samples were analyzed at the Hunan CDC laboratory. Detailed procedures can be found in published articles [20].

Surveillance of hantavirus infections among rodent hosts from 2006 to 2010 was conducted once per month for three consecutive nights. At least 300 medium-sized steel traps were set each night (baited with peanuts) and were recovered in the morning. More than 100 of these traps were placed indoors at approximately 12- to 15-meter intervals, and more than 200 traps were placed outdoors (every 5 meters in each row, with 50 meters between rows). A total of 698 rodents were captured out of 36,243 effective traps. “Relative rodent density”, used as an indicator of abundance, was calculated as the number of rodents captured, divided by the number of traps (Table 1).

**Table 1:** Data sources and illustration.

Data	Sources	Illustration
Patient data	Hunan Center for Disease Control and Prevention	Case reports
Rodent density	Hunan Center for Disease Control and Prevention	Monitoring reports
NDVI	International Scientific Data Service Platform	Remote sensing images
Temperature	China Meteorological Data Sharing Service System	Site data
Precipitation	China Meteorological Data Sharing Service System	Site data
Humidity	China Meteorological Data Sharing Service System	Site data
The urbanization rate	Hunan Statistics Yearbook	Statistics reports
GDP	Hunan Statistics Yearbook	Statistics reports

Meteorological data (monthly average temperature, monthly average relative humidity, and monthly precipitation) for the 2006 to 2010 period were obtained from the China Meteorological Data Sharing Service System (http://cdc.cma.gov.cn/index.jsp). GDP and the urbanization rate of the population were obtained from the Hunan Statistics Yearbook. Immigrant population data were obtained from Hunan Public Security Department. Data for the estimation of the NDVI was obtained from the International Scientific Data Service Platform (1 km spatial resolution; http://datamirror.csdb.cn/). The NDVI was generated from a transformation of the near infrared (NIR) and red wavelengths (RED) using the equation:
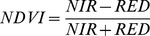
(1)


Land use data were obtained from the Second National Land Survey and were categorized as to cultivated land, forest, grass, or residential land. The data set was analyzed in ArcGIS 9.3 (ESRI Inc., Redlands, CA, USA) and included a digital map of Chenzhou (1∶50000), geocoding, case information, and population information. The data for each variable were converted to the same geographic projection and clipped to the study area.

### Ethical review

The present study was reviewed by the research institutional review board of the Hunan CDC. The review board determined that utilization of disease surveillance data did not require oversight by an ethics committee. Because the data were publicly available secondary data and were analyzed anonymously, no ethics statement was required for the work. The methods did not include animal experimentation, so it was not necessary to obtain an animal ethics license from the Animal Experiment Board. The species captured in this study were not protected wildlife and were not included in the China Species Red List.

### Data analysis

The principal component analysis was performed using the 2006–2010 data on natural factors (relative rodent density, NDVI, monthly average temperature, monthly rainfall and monthly average relative humidity) and social factors (GDP and urbanization rate). Four principal components that included three natural components (F1: rodent density, NDVI for rice paddies and temperature; F2: rainfall and relative humidity, and F3: rodent density and relative humidity) and one social component (F4: GDP and the urbanization rate of the population) were extracted.

Cross correlation analysis, adjusted for seasonality, was performed to infer the time-lag effects between variables. Each sequence of variables was filtered to convert it to white noise before proceeding with the cross correlation analysis. The correlation between the residual sequence of HFRS incidence and the residual sequences of the environmental variables (F1, F2, F3, F4), lagged 0∼6 months, was then calculated.

To confirm the correlation between lagged variables and HFRS incidence, the polynomial distributed lag (PDL) model with a lagged dependent variable was used to examine the contribution of various variables to HFRS incidence. The PDL model was:
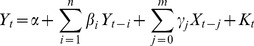
(2)

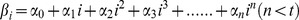
(3)

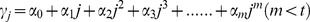
(4)where *Y* is the dependent variable, *α* is the regression coefficient of the independent variable, *n* and *m* are the lag phases, *β* and *γ* are the lagged regression coefficients, and *K* is the random disturbance term.

## Results

### Characteristics of HFRS epidemics

**Figure 2:**
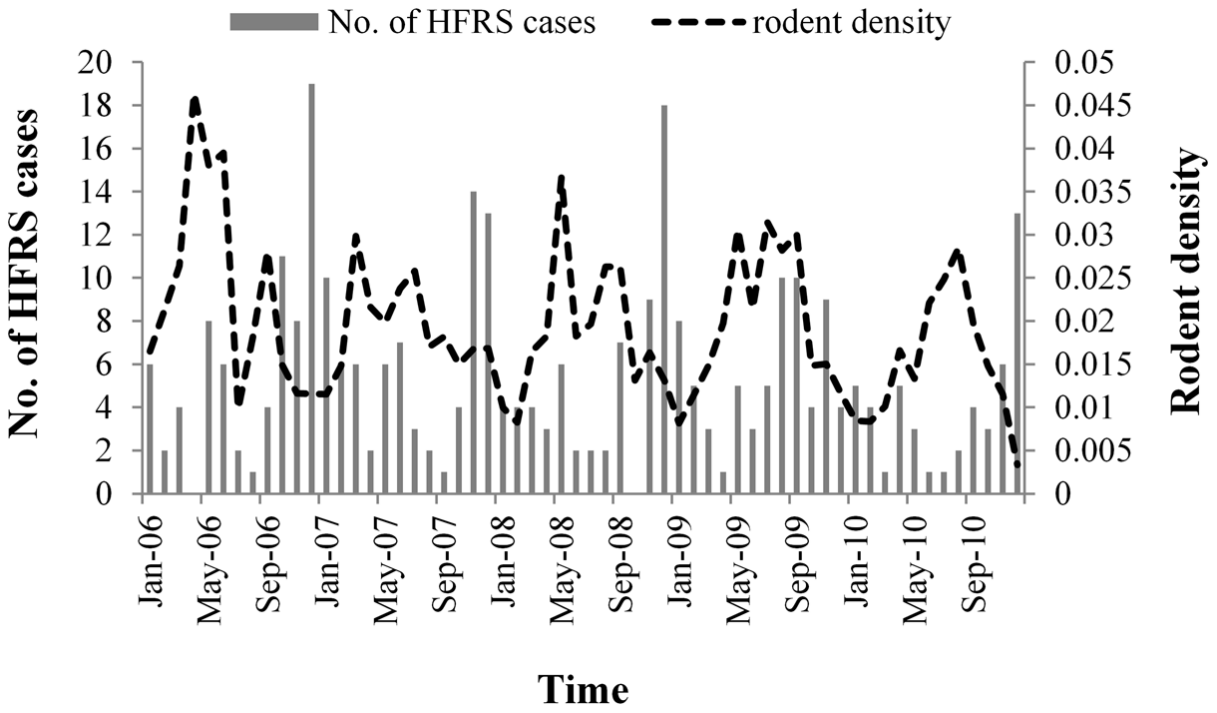
HFRS cases and rodent density.

A total of 321 HFRS cases were reported in Chenzhou, and yearly average HFRS incidence remained stable, during the study period. Yearly average HFRS was 1.53/100,000 (71 cases) in 2006, 1.59/100,000 (74 cases) in 2007, 1.29/100,000 (61 cases) in 2008, 1.41/100,000 (67 cases) in 2009, and 0.96/100,000 (48 cases) in 2010, Analysis of monthly HFRS cases revealed that HFRS incidence was higher from November to January and lower in March, April, July, and August (Figure 2).

A total of 251 rodents were captured at specific industry monitoring sites (catering industry and processing industry in Beihu and Suxian Districts). Captured rodents consisted mostly of the species *Rattus norvegicus*, *Rattus flavipectus*, and *Mus musculus*, which are known hosts of hantavirus [21]. The capture rate was 2.06 (per 100 trap-nights). A total of 125 rodents were captured in residential monitoring sites in Beihu District; the capture rate was 1.04 (per 100 trap-nights). A total of 322 rodents (mainly *R. flavipectus* and *M. musculus*) were captured at rural monitoring sites (Table 2). The capture rate was 2.68 (per 100 trap-nights). There was an annual peak of rodent density from April to September. The maximum capture rate was 4.64, which was recorded in April, 2006. The minimum capture rate was 0.81, recorded in January, 2009 (Figure 2).

**Table 2:** The number of rodents of each species captured, 2006–2010.

	*R.norvegicus*	*R.flavipectus*	*M.musculus*	*A.agrarius*	*R.rattoides*	Others species
**2006**	68	48	35	11	4	8
**2007**	48	24	56	9	1	0
**2008**	47	7	74	5	0	2
**2009**	39	7	93	5	0	1
**2010**	34	7	65	3	0	0

**Figure 3:**
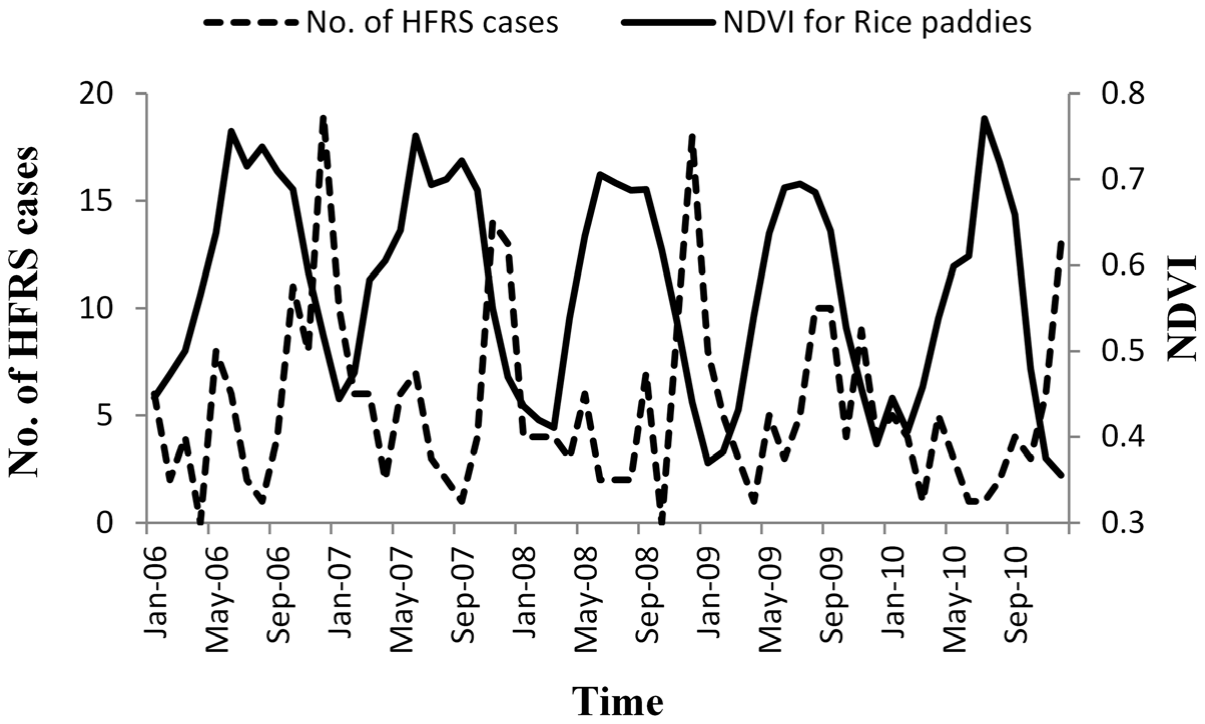
HFRS cases and normalized difference vegetation index (NDVI) for cultivated land.

**Figure 4:**
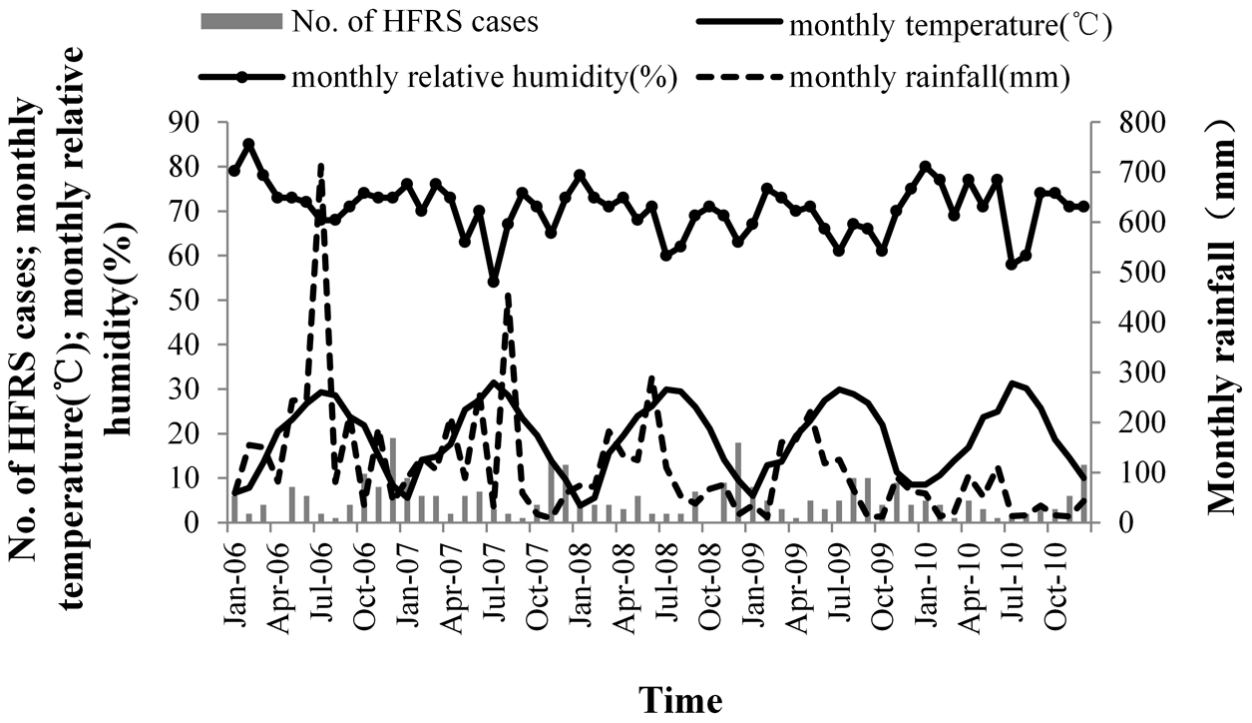
HFRS cases and meteorological factors.

The monthly NDVI for cultivated land ranged between 0.3 to 0.8. The NDVI increased from January to July, and then decreased each month after the peak of variation from August to October. The peak HFRS incidence was preceded by the peak NDVI and the peak monthly average temperature and monthly average rainfall, with a 3∼4 month lag (Figure 3, Figure 4).

The results of the analysis of the relationship between HFRS incidence and socioeconomic factors (GDP and urbanization rate) indicated that the urbanization rate was significant negatively correlated with HFRS incidence (r = −0.903, *P*<0.001) and GDP (r = −0.627, *P*<0.05). HFRS incidence declined as urbanization rate and GDP increased.

### Principal component analysis

The results of the principal component analysis revealed that component 1 (F1), component 2 (F2) and component 3 (F3) accounted for 91.66% of the total variability in natural factors. F1 was closely associated with rodent density, NDVI, and temperature. F2 was closely associated with rainfall and humidity. F3 was closely associated with rodent density and humidity (Table 3). Component 4 (F4) accounted for 85.58% of the total variation in socioeconomic factors (Table 3).

**Table 3:** Coefficients for the relationship between principal components and variables.

Principal components	Rodent density	NDVI	Temperature	Precipitation	Relative humidity	Urbanization rate	GDP
**F1**	0.421	0.556	0.572	0.207	−0.378		
**F2**	0.059	0.079	−0.052	0.681	0.555		
**F3**	0.759	0.015	−0.946	−0.400	0.504		
**F4**						0.574	0.608

### Association between HFRS incidence and variables

The correlation between HFRS incidence, the variables, the three natural components (F1, F2, F3), and the socioeconomic component (F4) were calculated with a lag of 0∼6 months. Monthly HFRS incidence was positively correlated with rodent density with a 6-month lag (r = 0.354, *P = *0.009). HFRS incidence was preceded by NDVI (r = 0.49, *P<*0.001), temperature (r = 0.515, *P*<0.001) and rainfall (r = 0.414, *P = *0.002), with a 5-month lag (Table 4). Monthly HFRS incidence was correlated with F1 (rodent density, NDVI for rice paddies and temperature; r = 0.179, *P = *0.192) and F2 (rainfall and relative humidity; r = −0.289, *P = *0.032), with a 4-month lag; HFRS incidence was preceded by F3 (rodent density and relative humidity) with a 5-month lag (r = −0.523, *P<*0.001). HFRS incidence was correlated with F4 (GDP and urbanization rate of population; r = −0.376, *P* = 0.003) (Table 4).

**Table 4:** Cross-correlation coefficients of the variables and notifications of HFRS.

Variables	Lag 0	Lag 1	Lag 2	Lag 3	Lag 4	Lag 5	Lag 6
**rodent density**	−0.173	−0.114	−0.076	0.092	0.256	0.127	0.354**
**NDVI**	−0.277	−0.048	0.227	0.431	0.476	0.490**	0.379
**temperature**	−0.388	−0.136	0.11	0.33	0.475	0.515**	0.417
**rainfall**	−0.23	−0.217	−0.153	0.247	0.162	0.411**	0.396
**relative humidity**	−0.01	0.123	0.181	0.247*	0.13	0.07	−0.122
**F1**	0.002	0.131	−0.087	−0.102	0.179	−0.166	0.125
**F2**	−0.229	−0.223	−0.089	0.239	−0.289*	0.153	0.101
**F3**	0.204	0.045	0.093	−0.054	−0.077	−0.523**	−0.136
**F4**	−0.376**	−0.114	−0.221	−0.283*	0.088	−0.141	−0.069

*P<0.05,*

*P<0.01.*

### The PDL model

The PDL model yielded the best fit based on the R-squared and AIC (Akaike information criterion). First, three principal components based on natural factors were used to build Model 1 (R^2^ = 0.656, AIC = 5.023). Socioeconomic factors (F4) were included in Model 2 (R^2^ = 0.677, AIC = 5.106). Finally, 2nd-order autoregression was considered in Model 3, which indicated that the number of notified HFRS infections in the current month was related to the numbers of cases occurring in the previous 1 and 2 months (R^2^ = 0.857, AIC = 4.799).

**Figure 5:**
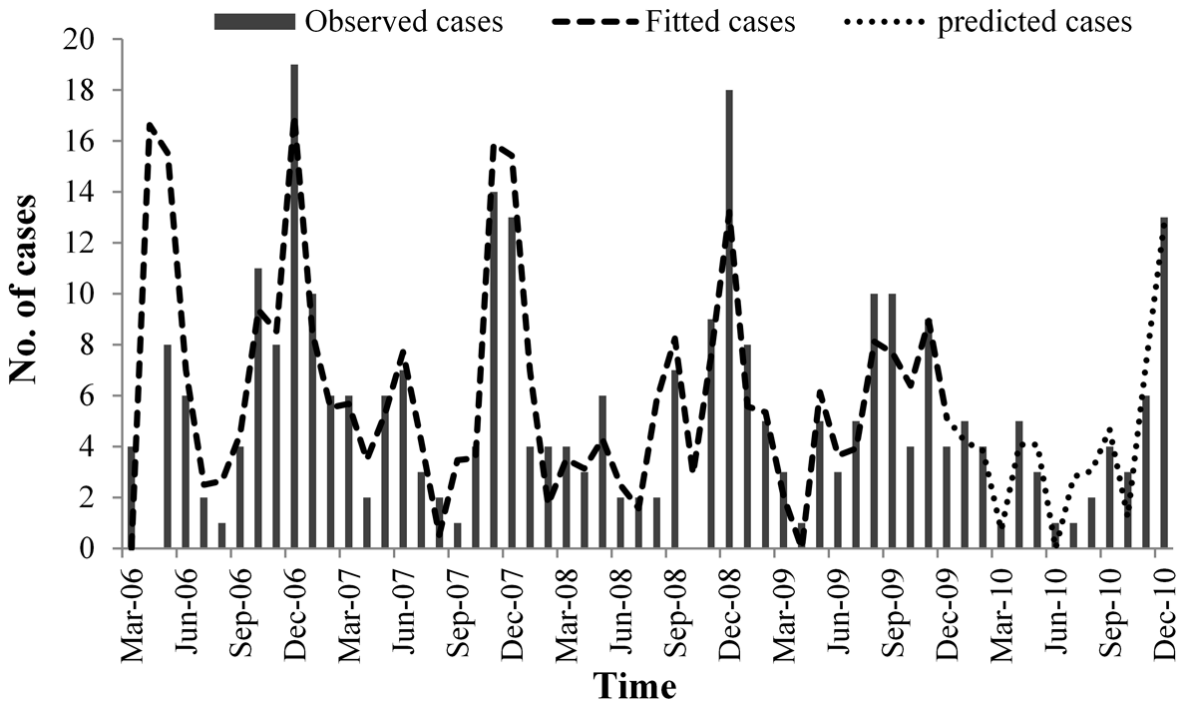
Fitted result of the optimal model.

The results of the optimal model (Model 3) indicated that HFRS incidence was affected not only by the natural factors but also by the socioeconomic factors (Table 5). In addition, monthly HFRS incidence was strongly autocorrelated. The estimated/expected number of cases from the regression model fits very well to the observed number of HFRS cases, including the peak values (Figure 5).

**Table 5:** Fitted results of the model.

	MODEL-1			MODEL-2				MODEL-3					
	F1	F2	F3	F1	F2	F3	F4	F1	F2	F3	F4	lag-1	lag-2
Constant term	0.664	0.050	0.692	0.539	−0.080	0.776	−2.267	−0.012	−0.017	0.427	−0.268	0.214	0.338
Linear coefficient	0.227	−0.830	0.928	0.076	−1.029	0.952	0.612	−0.259	−1.042	0.889	5.950	−0.455	0.028
Quadratic coefficient	0.118	0.741	0.815	−0.002	0.502	0.825	−0.619	−0.034	0.732	0.892	−2.077		−0.283
Cubic coefficient	0.337	1.910	0.355	0.307	1.712	0.395	2.004	0.665	2.045	0.435	−3.800		
R^2^	0.656			0.677				0.857					
AIC	5.023			5.106				4.799					
RMSE	2.441			2.366				1.573					

## Discussion

To the best of our knowledge, little is known about the combined effect of environmental variations (animal reservoirs, natural and socioeconomic factors) on the transmission and persistence of HFRS. In general terms, rodent density and the extent of high-risk behaviors depend on natural factors. Changes in the animal reservoir may lead to emergence of new epidemics, and threats to human health. However, economic development may improve the residential environment, which could inhibit disease transmission from rodent vectors to humans through decreased contact.

In our analysis, the optimal model revealed that HFRS incidence was positively correlated with rainfall and relative humidity in Chenzhou. Rainfall is an important factor in HFRS morbidity, because increased rainfall provides better growth conditions for vegetation that directly or indirectly provides rodents with food, which leads to increases in rodent populations [13]. There is also a very close association between wet or very humid habitat types, and rodent population size [22], [23], because the moist environment provides suitable conditions [21]. HFRS epidemic areas are mostly distributed in low-lying moist regions or sub-humid regions [12], [21]. From 2006 to 2010, monthly average relative humidity ranged between 60% to 85%, which is conducive for the transmission HFRS. The maximum average relative humidity occurred in January and February. The minimum average relative humidity was in July and August.

Temperature and NDVI were important factors for HFRS epidemics and were positively associated with HFRS incidence. Temperature can affect rodent pregnancy rate, litter size, birth rate, and survival rate, and is an important factor in the fluctuation of rodent population size [24]. Rodent survival is greater during warmer winters than in colder winters, which leads to greater rodent population densities [25], [26]. There was a peak in temperature from June to August, and the peaks in HFRS cases were in May and June, and from November to January, indicating that HFRS incidence lagged behind temperature by approximately 4∼5 months. Temperature can also directly affect the geographic distribution of rodents, because they prefer warmer areas. Temperature is positively correlated with vegetation, which provides food for rodents. Thus, rodent density directly and indirectly depends on the local temperature [27]. Temperature ranged between 5°C to 30°C during the study period, which may have increased the rodent population size and indirectly increased HFRS morbidity in Chenzhou. The NDVI reflects the level of vegetation coverage [28], which is a good indicator of food and living conditions for rodents. It is correlated with the amount and productivity of vegetation and crops. Most rodent species responded directly to fluctuations in food availability, and population densities are driven by changes in food resources [29], [30]. Vegetation also provides shelter and safety (e.g., from predators).

HFRS incidence was positively correlated with rodent density. This result indicates that fluctuations in rodent populations had an important effect on HFRS incidence. Rodent population density peaked in March, April, August, and September. The peaks in HFRS cases were in May and June, and from November to January, indicating that HFRS incidence lagged behind rodent density by approximately 2∼3 months. Hantavirus infection rates can increase with increased rodent density if the infected rodents increase their contact with humans [31]–[33]. Therefore, our results suggest that rodent density fluctuations could be used to forecast changes in HFRS incidence and that HFRS transmission could be controlled by reducing the number of rodents in residential areas.

HFRS incidence was negatively correlated with GDP (r = −0.627) and the urbanization rate (r = −0.903). HFRS incidence decreased with the increase in per capita GDP and urbanization rate. These results suggest that economic development may reduce HFRS transmission, which is consistent with the findings of a previous study [13]. Rodent density decreases with the development of economy and culture. It is generally low in developed countries. Rodent density is lower in the developed areas than in less developed areas in China [34]. Economic development has led to improvements in living conditions, because the size of polluted, disorganized, and poor areas have been reduced. Meanwhile, there have been improvements in deratization methods and in public awareness about rodent prevention and control. Variation in the ecology of the environment that results from extensive construction will certainly have direct or indirect effects on the living conditions and food for rodents, thus leading to variation in disease transmission intensity [35]. There has been increased awareness of diseases prevention and control measures as health care services have improved. However, the environment of most villages in China is suitable for rodent survival and development, and high rodent densities persist in many fields and villages [34]. Peasants work long hours in areas where rodents are active, and increased contact with the animals' secretions (e.g. feces, urine and saliva) means that farmers are the main high risk group for HFRS [4]. People in rural areas come into contact with rats more frequently than in urban areas, and the large number of rural residents migrating into cities may be another explanation for the year-by-year decrease in HFRS. Pollution of the fields and other vegetation areas by fertilizers, pesticides, and heavy metals also affects the living conditions and food availability for rodents, and the toxic effects of these substances has a negative effect on population growth [36].

Compared to our previous work in Changsha [11], this analysis indicates many similarities between these two areas. HFRS was positively correlated with rodent density and the NDVI, and was influenced by temperature and rainfall in Chenzhou and Changsha. This similarity is likely a reflection of comparable host behaviors and habitats . The primary difference between this study and the Changsha study is that we incorporated socioeconomic factors into this analysis. Few studies are currently examining the relationship between HFRS incidence and socioeconomic factors. In this study, we comprehensively considered the effects of natural and socioeconomic factors on HFRS incidence. The addition of socioeconomic variables improved the model fit. Models based on natural and social variables had better performance (R^2^ = 0.677) than models based on only natural variables (R^2^ = 0.656). In this study, the PDL model used component variables, which reduced the effect of higher order multicollinearity among variables and improved the fit.

There were some limitations of this study. First, the monthly average temperature, which was measured in the air, was different from the surface temperature. Surface temperature has a more direct effect on rodents, so it would be more informative to incorporate surface temperature into models of HFRS incidence. Second, HFRS cases were from a passive, instead of active, surveillance system, so some cases may not have been identified. Patients with less serious or less obvious symptoms may not seek medical care, which would result in an underestimate of the true incidence. Finally, the effects of extreme weather conditions (e.g., high temperature, torrential rain, and drought) on the survival and reproduction of rodents, and on HFRS transmission, needs further study. Furthermore, this was a population-level study, and the potential of the ecological fallacy to affect the results is unavoidable in a study of this kind.

In conclusion, changes in the risk of HFRS may be the result of changes in contact between humans and the rodent reservoir, which are caused by changes in natural and socioeconomic factors. The results of our analysis provide theoretical support for this hypothesis and indicate that further study of variation in HFRS incidence would be beneficial for the prevention and control of this disease.

## Supporting Information

Checklist S1 **STROBE checklist.**
(DOC)
